# Calcium Hydroxide Combined with Bioactive Glasses: Physicochemical, Antimicrobial and Bond Strength Evaluation

**DOI:** 10.1590/0103-644020267120

**Published:** 2026-10-19

**Authors:** Larissa Braz Pontes, Gabriel Dutra Rissato, Pedro Luís Busto Rosim, Airton Oliveira Santos-Junior, Marina Trevelin Souza, Edgar Dutra Zanotto, Mário Tanomaru-Filho, Juliane Maria Guerreiro-Tanomaru

**Affiliations:** 1 Department of Restorative Dentistry, School of Dentistry, São Paulo State University (UNESP), Araraquara, São Paulo, Brazil; 2 Department of Dentistry, Universidade da Amazônia (UNAMA), Belém, Pará, Brazil; 3 Department of Materials Engineering, Vitreous Materials Laboratory (LaMaV), Federal University of São Carlos(UFSCar), São Carlos, São Paulo, Brazil

**Keywords:** Calcium hydroxide, glass, bioactive, copper, endodontics

## Abstract

This study evaluated the pH, solubility, antimicrobial activity, and bond strength of a calcium hydroxide intracanal medication (80% calcium hydroxide and 20% zirconium oxide in polyethylene glycol 400 vehicle), evaluated alone or combined with 20% F18 bioactive glass or copper-doped F18 bioactive glass. Polyethylene tubes containing the medications were immersed in distilled water. pH (n=10) was measured after 1, 3, 7, 14, and 21 days, and solubility (n=7) was determined after 7 and 14 days. Antimicrobial activity (n=6) was assessed using a modified direct contact test against *Enterococcus faecalis* and/or *Candida albicans*. For bond strength analysis (n=12), bovine roots were filled with the medications for 14 days and subsequently obturated with Bio-C Sealer or AH Plus. Specimens were subjected to a push-out test, and data were analyzed by ANOVA and Tukey’s test (α=0.05). All formulations exhibited an alkaline pH (p>0.05). The formulation without bioactive glass showed higher solubility (p<0.05). The copper-doped bioactive glass formulation was the most effective against *E. faecalis* in single- and dual-species biofilms (p<0.05), whereas the formulation without bioactive glass completely eradicated *C. albicans* (p<0.05). Regarding bond strength, no differences were observed among groups obturated with AH Plus (p>0.05). When Bio-C Sealer was used, the F18 bioactive glass formulation exhibited the highest bond strength to dentin (p<0.05). Calcium hydroxide combined with F18 bioactive glass reduced solubility and improved the bond strength of Bio-C Sealer, whereas copper-doped bioactive glass enhanced antimicrobial performance, supporting their potential clinical applicability.

## Resumo

Este estudo avaliou o pH, a solubilidade, a atividade antimicrobiana e a resistência de união de uma medicação intracanal de hidróxido de cálcio (80% de hidróxido de cálcio e 20% de óxido de zircônio e veículo polietilenoglicol 400), testada isoladamente ou associada a 20% de biovidro F18 ou biovidro F18 dopado com cobre. Tubos de polietileno contendo as medicações foram imersos em água destilada. O pH (n=10) foi medido após 1, 3, 7, 14 e 21 dias, e a solubilidade (n=7) foi determinada após 7 e 14 dias. A atividade antimicrobiana (n=6) foi avaliada por meio de teste de contato direto modificado contra *Enterococcus faecalis* e/ou *Candida albicans*. Para a análise da resistência de união (n=12), raízes bovinas foram medicadas por 14 dias e posteriormente obturadas com Bio-C Sealer ou AH Plus. Os espécimes foram submetidos ao teste de push-out, e os dados analisados por ANOVA e teste de Tukey (α=0,05). Todas as formulações apresentaram pH alcalino (p>0,05). A formulação sem biovidro apresentou maior solubilidade (p<0,05). A formulação com biovidro F18 dopado com cobre foi a mais eficaz contra *E. faecalis* em biofilmes mono e multiespécies (p<0,05), enquanto a formulação sem biovidro erradicou completamente *C. albicans* (p<0,05). Quanto à resistência de união, não houve diferenças entre os grupos obturados com AH Plus (p>0,05). Quando o Bio-C Sealer foi utilizado, a formulação com biovidro F18 apresentou maior resistência de união à dentina (p<0,05). A associação do hidróxido de cálcio ao biovidro F18 reduziu a solubilidade e aumentou a resistência de união do Bio-C Sealer, enquanto o biovidro F18 dopado com cobre melhorou o desempenho antimicrobiano.

**Figure:**
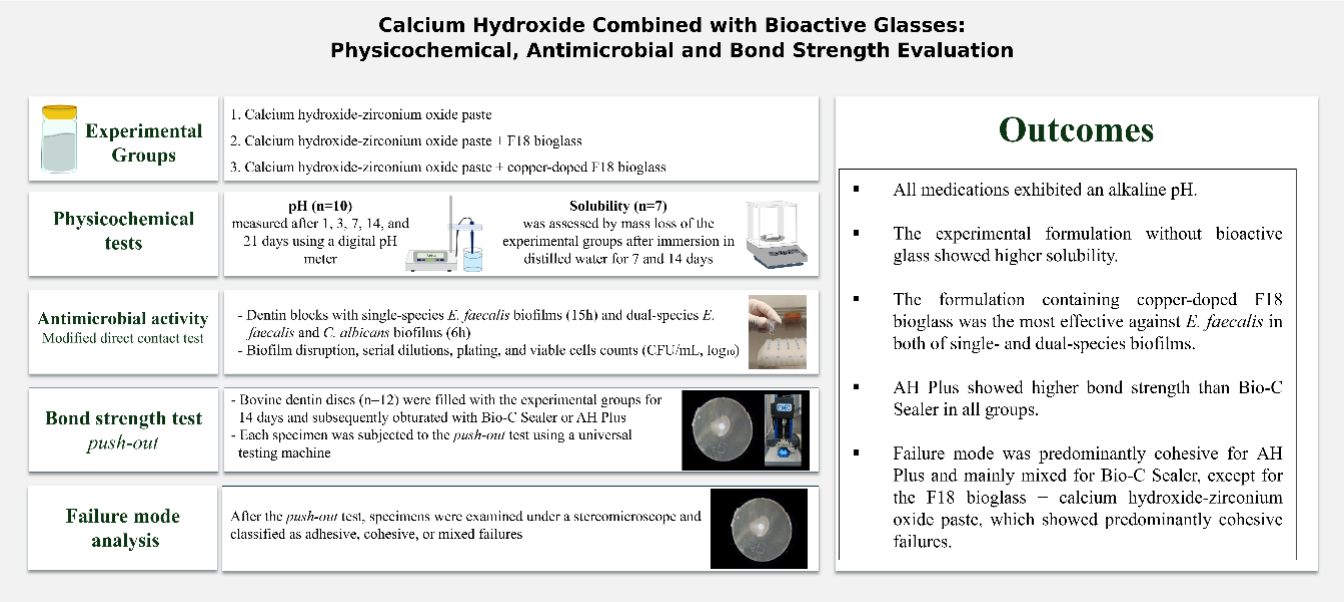

## Introduction

Disinfection of the root canal system is essential for successful endodontic treatment and repair of periapical tissues ^1^. Intracanal medication contributes to the elimination of persistent microorganisms after root canal preparation ^1^. Calcium hydroxide is widely used due to its antimicrobial and biological properties ^2^. Its mechanism of action is associated with the release of hydroxyl ions, increasing pH, preventing bacterial growth and promoting tissue repair ^2^. However, despite its recognized antimicrobial activity, calcium hydroxide presents limited efficacy against resistant microorganisms such as *E. faecalis*, especially when organized in mature biofilms ^1^ ^2^.

Despite these benefits, the effectiveness of calcium hydroxide depends on the formulation of the medication, including the vehicle and the presence of additives ^2^. Polyethylene glycol 400 is a viscous vehicle that allows continuous release of hydroxyl ions, thereby prolonging the antimicrobial effect of calcium hydroxide ^2^. The incorporation of radiopacifiers, such as zirconium oxide (ZrO₂), ensures radiographic detection without compromising biocompatibility or antimicrobial activity, while maintaining alkaline pH and favoring calcium ion release ^3^ ^4^ ^5^. The addition of 20% of ZrO₂ to Portland cement provides adequate radiopacity and increases bond strength to dentin ^6^, whereas concentrations of 30% in calcium silicate-based cements promote hydroxyapatite formation and bioactivity ^7^. These advances indicate that modifying the formulation of calcium hydroxide with different vehicles and additives may enhance its antimicrobial performance and physicochemical properties, offering new perspectives for calcium hydroxide-based intracanal medications.

New formulations have been developed using bioactive materials, such as bioactive glass, due to their ability to interact with biological tissues and promote hard tissue regeneration ^8^. The combination of bioactive materials, such as the Bio-C Temp bioceramic intracanal medication, with calcium hydroxide-based formulations to increase antimicrobial effectiveness has been demonstrated ^9^. F18 bioactive glass (Vetra Biomaterials Ltda., Ribeirão Preto, SP, Brazil) developed by the Vitreous Materials Laboratory (LaMaV), Department of Materials Engineering, Federal University of São Carlos (UFSCar, São Carlos, SP, Brazil), composed of silicon dioxide, calcium oxide, sodium oxide, magnesium oxide, potassium oxide, and phosphorus pentoxide, stimulates cell proliferation and hydroxyapatite formation, thereby promoting bone mineralization and repair of mineralized structures ^10^. Its antimicrobial mechanism is associated with increased pH, osmotic pressure, and interaction with the cell wall ^11^. F18 bioactive glass demonstrated biocompatibility and stimulation of biomineralization as an intracanal medication mixed with distilled water, but lower effectiveness against *E. faecalis* compared with calcium hydroxide ^10^. Final irrigation with cobalt-doped bioactive glass solution favored collagen maturation and the expression of cell markers in regenerative endodontic protocols ^12^. The association of F18 bioactive glass in calcium hydroxide-based formulations may represent a strategy for formulating intracanal medication with antimicrobial and bioactive properties.

Copper-doped bioactive glass has recently been developed to enhance its antimicrobial and biological properties ^13^. This modification increases antimicrobial efficacy against both Gram-positive and Gram-negative microorganisms compared with conventional F18 bioactive glass ^14^. Copper induces the production of reactive oxygen species, promoting oxidative stress and damage to microbial cells ^14^. Furthermore, copper can induce membrane instability and metabolic imbalance in fungi, compromising microbial viability ^15^. In addition, copper-doped bioactive glass demonstrates cytocompatibility, induces mineralized tissue formation, and reduces the expression of pro-inflammatory interleukins, thereby exhibiting bioactive and osteogenic potential ^13^. These findings indicate that copper-doped bioactive glass represents an advance over conventional F18 bioactive glass, combining antimicrobial and biological properties that justify its investigation in Endodontics.

Considering the known properties of calcium hydroxide as an intracanal medication and the bioactive and antimicrobial potential of F18 bioactive glass and copper-doped bioactive glass, it is relevant to investigate the effect of these combinations in experimental formulations. Therefore, the aim of this study was to evaluate the physicochemical properties, antimicrobial activity, and bond strength of calcium hydroxide pastes combined with F18 bioactive glass or copper-doped bioactive glass. The null hypothesis was that these incorporations would not alter any of the evaluated properties.

## Materials and methods

Table 1 summarizes the experimental groups, including materials and manufacturers, composition, and proportions. This laboratory study was conducted and reported in accordance with the Preferred Reporting Items for Laboratory Studies in Endodontology (PRILE) 2021 guidelines ^16^. The corresponding PRILE flowchart is shown in Figure 1.

**Table 1:** Experimental groups, materials and manufacturers, composition, and proportions

Group	Materials and manufacturers	Composition	Proportion (%)
CH-EXP	Calcium hydroxide (Merck/Sigma-Aldrich, Barueri, SP, Brazil); zirconium oxide; polyethylene glycol 400 (vehicle)	Calcium hydroxide, zirconium oxide; vehicle: polyethylene glycol 400 (PEG 400)	80% calcium hydroxide + 20% zirconium oxide; PEG 400
CH-F18BG	Calcium hydroxide (Merck/Sigma-Aldrich, Barueri, SP, Brazil); F18 bioactive glass (Vetra Biomaterials Ltda., Ribeirão Preto, SP, Brazil); zirconium oxide; polyethylene glycol 400.	Calcium hydroxide, zirconium oxide; vehicle: PEG 400; F18 bioactive glass (SiO₂, CaO, Na₂O, MgO, K₂O, P₂O₅)	80% CH-EXP + 20% F18 bioactive glass; PEG 400
CH-Cu/F18BG	Calcium hydroxide (Merck/Sigma-Aldrich, Barueri, SP, Brazil); copper-doped F18 bioactive glass (Vetra Biomaterials Ltda., Ribeirão Preto, SP, Brazil); zirconium oxide; polyethylene glycol 400.	Calcium hydroxide, zirconium oxide; vehicle: PEG 400; copper-doped F18 bioactive glass (SiO₂, CaO, Na₂O, MgO, K₂O, P₂O₅, CuO)	80% CH-EXP + 20% copper-doped F18 bioactive glass; PEG 400

CH-EXP, experimental calcium hydroxide paste; CH-F18BG, calcium hydroxide + F18 bioactive glass; CH-Cu/F18BG, calcium hydroxide + copper-doped F18 bioactive glass; PEG 400, polyethylene glycol 400. Chemical formulas: SiO₂, silicon dioxide; CaO, calcium oxide; Na₂O, sodium oxide; MgO, magnesium oxide; K₂O, potassium oxide; P₂O₅, phosphorus pentoxide; CuO, copper oxide.

The concentration of zirconium oxide was defined according to a previous evaluation of the radiopacity of the formulations using different proportions (10 and 20% by weight). The concentration of 20% by weight was considered sufficient to provide adequate radiopacity. The incorporation of 20 wt% (weight percentage) of F18 bioactive glass was based on preliminary studies that identified this concentration as optimal for promoting ion release and inducing hydroxyapatite formation without compromising the material’s handling properties ^9^.

### Preparation of bioactive glass particles

F18 Bioglass and the doped glass (F18-CuO) were synthesized by mixing the precursor oxides, including silica, calcium oxide, phosphorus oxide, and sodium oxide, followed by melting at 1450°C in a platinum crucible for 3 h ^10^. After melting, the material was rapidly quenched in water to form a frit and subsequently dried at 100°C for 24 h. The material was then ground in a planetary mill (Pulverisette, Fritsch, Germany) until a mean particle size of approximately 5 μm was obtained. Particle size distribution was analyzed by laser diffraction (Horiba LA-930, Japan), revealing particles ranging from 0.1 to 80 μm ^10^.

### pH

Polyethylene tubes (n=10), measuring 10 mm in length and 1.6 mm in internal diameter, were filled with the medications. The size of the tubes was determined by previous studies using a similar methodology (9,17). Each tube was immersed in a plastic container with 10 mL of distilled water and stored in an incubator at 37 °C. The pH of the immersion solution was measured at the end of each experimental period (1, 3, 7, 14, and 21 days) at room temperature (25 °C), using a previously calibrated digital pH meter (FiveEasy Plus FP20, Mettler Toledo®, Columbus, OH, USA). Each sample was analyzed in triplicate, and the mean values were considered for statistical analysis. After each measurement, the solution used for reading was discarded and replaced with the same volume of fresh distilled water. Containers with distilled water only were used as controls.

**Figure 1:**
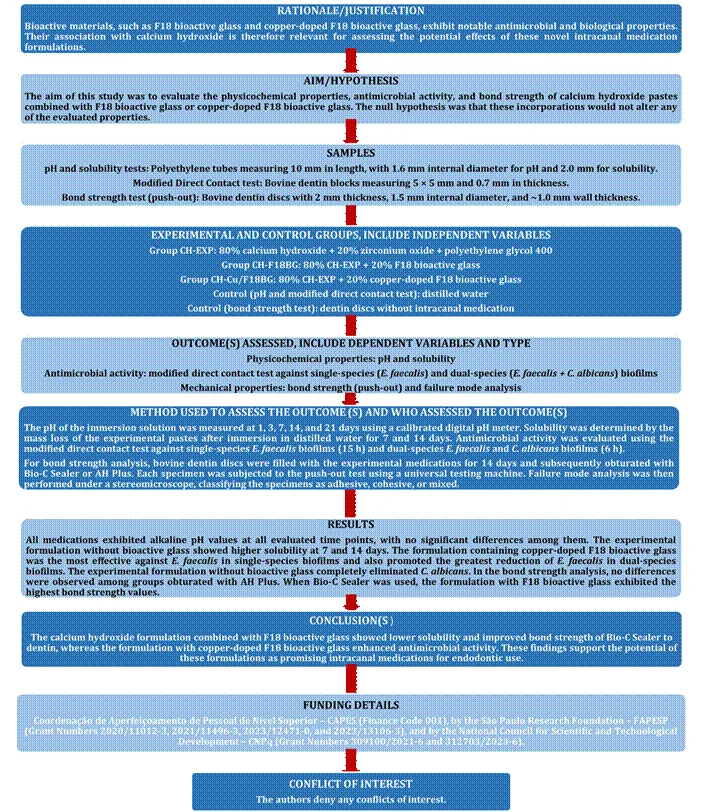
Flowchart following the Preferred Reporting Items for Laboratory Studies in Endodontology (PRILE) 2021 guidelines.

### Solubility

Polyethylene tubes (n=7), measuring 10 mm in length and 2 mm in internal diameter, were filled with the medications and stored in an incubator at 37 °C. The sample size was based on a previous study (9,17). After 24 h, each tube was weighed on an electronic analytical balance (OHAUS Adventurer®, New Jersey, USA) to determine the initial mass. The tubes were then immersed in plastic containers with 7.5 mL of distilled water at 37 °C. After 7 and 14 days, the samples were removed, dried in a vacuum desiccator, and reweighed until mass stabilization. Solubility was determined by mass loss, calculated as the difference between the initial and final values ^9^ ^17^.

### Antimicrobial activity - Modified direct contact test

The modified direct contact test was performed based on previous studies ^20^. For this assay, bovine dentin blocks (5 × 5 mm in width, 0.7 mm in thickness) were obtained from bovine central incisors using a precision cutting machine (Isomet 100 - Buehler, USA). The blocks (n=6) were then stored in test tubes containing distilled water and sterilized in an autoclave at 120 °C for 20 min. These blocks served as substrates for biofilm formation.

For single-species biofilm cultivation, the dentin blocks were immersed in 2 mL of Tryptic Soy Broth (BD-Difco) supplemented with glucose, containing 1% inoculum of *E. faecalis* (1 × 10⁸ CFU mL⁻¹). For dual-species biofilm cultivation, RPMI-1640 medium (Gibco) was used, in which the blocks were immersed together with a combined inoculum of *E. faecalis* (ATCC 29212) and *C. albicans* (ATCC 10231), with its purity assessed.

The biofilms were cultured in 24-well plates and maintained in a benchtop incubator with orbital agitation (Model 0816M28, Quimis Aparelhos Científicos LTDA, Brazil) at 37 °C and 80 rpm. The single-species biofilm was developed under microaerophilic conditions for 7 days ^18^, with complete medium replacement every 48 h. The dual-species biofilm was cultivated under aerobic conditions for 5 days ^19^, with daily medium replacement.

The antimicrobial assay was carried out using eluates of the tested pastes (50mg/mL in distilled water), prepared in an incubator at 37 °C for 48 h. After this period, the dentin blocks containing biofilm were washed with phosphate-buffered saline (PBS) to remove non-adherent cells. Each block was then immersed in a 500 µL Eppendorf tube containing 350 µL of the respective filtered eluate. Sterile distilled water was used as the control. The contact time with the eluates was 15 h for the single-species biofilm and 6 h for the dual-species biofilm, both maintained at 37 °C. Different exposure times were used based on pilot studies and the microbiological characteristics of each experimental model.

After the contact period, the blocks were transferred to Eppendorf tubes containing 1 mL of sterile saline solution and glass beads. The tubes were vortexed for 1 min to promote microbial suspension. Serial decimal dilutions were prepared, and three aliquots of 20 µL from each dilution were plated onto Petri dishes containing different culture media: Tryptic Soy Agar (BD-Difco) for the *E. faecalis* single-species biofilm; M-Enterococcus Agar (BD-Difco) and Sabouraud Dextrose Agar with Chloramphenicol (BD-Difco) for the *E. faecalis* and *C. albicans* dual-species biofilm. The plates were incubated at 37 °C for 48 h, and colony-forming units (CFU/mL) were subsequently counted. The results were converted into logarithmic values (log₁₀) prior to statistical analysis.

### Bond strength (push-out test)

The bond strength of the root canal sealers to bovine dentin previously exposed to the intracanal medications was evaluated using extracted bovine teeth. Sample size calculation was performed with G*Power software (version 3.1.7 for Windows, Heinrich Heine University, Düsseldorf, Germany). For this analysis, ANOVA was considered with an alpha error of 0.05 and a statistical power (1 - β) of 0.90. The effect size (1.675) was defined based on a previous study that employed a similar methodology ^21^. Based on this calculation, it was determined that 12 specimens per group represented the required sample size to detect statistically significant differences among groups.

After sample size determination, bovine maxillary incisors were selected. The crowns were removed, and the apical 2 mm were sectioned. The roots were embedded in resin and, after setting, were transversely sectioned with a precision cutting machine (Isomet 1000, Buehler Ltda, Lake Bluff, IL, USA) to obtain specimens with 2 mm thickness. Root canal preparation was standardized using a #702 tapered carbide bur mounted in a parallelometer, ensuring complete penetration of the root canal and producing uniform cylinders with an internal diameter of 1.5 mm and a wall thickness of approximately 1.0 mm. During preparation, the canals were irrigated with 1% sodium hypochlorite (NaOCl). Final irrigation consisted of immersion of the specimens in 2.5% NaOCl solution for 3 min, followed by 17% ethylenediaminetetraacetic acid (EDTA) (Biodinâmica, Indaiatuba, SP, Brazil) for 3 min, and finally in distilled water for 3 min. The canals were then filled with the intracanal medications (Table 1) and stored for 14 days at 37 °C in contact with gauze moistened with buffered distilled water.

After the period of contact with the intracanal medications, they were removed by irrigation with 5 mL of 1% NaOCl, followed by immersion in 17% EDTA with ultrasonic agitation (Digital Ultrasonic Bath, Cristófoli, PR, Brazil) for 3 min, and a final irrigation with 5 mL of distilled water. The canals were then dried with absorbent paper points (Tanari, Manaus, AM, Brazil). Subsequently, the specimens were obturated with the bioceramic sealer Bio-C Sealer (lot 74477; Angelus, Londrina, PR, Brazil) or the epoxy resin-based sealer AH Plus (lot 2410000527; Dentsply, Germany) and stored in an incubator at 37 °C under controlled relative humidity for 7 days.

Bond strength (push-out) was evaluated using a universal testing machine (Emic DL 2000, Instron, São José dos Pinhais, PR, Brazil) equipped with a 100 N load cell and operating at a constant speed of 0.5 mm/min. A stainless steel cylindrical plunger with a 1.3 mm diameter was positioned to apply continuous load until displacement of the root canal filling material occurred. The maximum load recorded (Newton, N) was converted into bond strength (megapascal, MPa) using the formula MPa = N / A, where *N* is the maximum load in Newtons and *A* is the adhesion surface area (square millimeters, mm²), calculated by the equation A = 2πrh, with π = 3.14, *r* corresponding to the standardized root canal radius, and *h* representing the thickness of each section in millimeters, measured with a digital caliper (Mitutoyo, Suzano, SP, Brazil).

### Failure mode classification

After the push-out tests, the specimens were examined under a stereomicroscope to determine the failure mode. This qualitative evaluation allowed the specimens to be classified into three categories: adhesive failure, characterized by the absence of sealer on the dentin walls; cohesive failure, characterized by the presence of sealer firmly adhered to the dentin walls; and mixed failure, when both patterns were observed.

### Statistical analysis

Data normality and homogeneity of variances were assessed using the Shapiro-Wilk and Levene tests, respectively. Statistical significance was analyzed via one-way ANOVA, followed by Tukey’s post hoc test for multiple comparisons. The significance level was set at 5%.

## Results

Table 2 presents the results of pH and solubility tests. All experimental formulations, calcium hydroxide paste either alone or combined with F18 bioactive glass or copper-doped F18 bioactive glass, promoted significant alkalinization of the medium at all evaluated periods. No differences were observed among them (p>0.05), but all showed higher values than the control (p<0.05). Regarding solubility, the calcium hydroxide-based experimental paste exhibited greater mass loss at 7 and 14 days (p<0.05), whereas the formulations containing F18 bioactive glass or copper-doped F18 bioactive glass showed lower solubility and did not differ from each other (p>0.05).

**Table 2:** Mean and standard deviation (±SD) of pH values and solubility (% mass loss) in the experimental groups

Period	CH-EXP	CH-F18BG	CH-Cu/F18BG	Control
pH			
1 day	11.69 ± 0.264^a^	11.56 ± 0.157^a^	11.42 ± 0.363^a^	7.678 ± 0.145^b^
3 days	10.98 ± 0.555^a^	10.94 ± 0.579^a^	10.86 ± 0.409^a^	7.692 ± 0.157^b^
7 days	11.15 ± 0.37^a^	10.76 ± 0.29^a^	10.85 ± 0.483^a^	7.630 ± 0.106^b^
14 days	10.58 ± 0.252^a^	10.36 ± 0.283^a^	10.52 ± 0.162^a^	7.646 ± 0.235^b^
21 days	9.835 ± 0.402^a^	9.843 ± 0.706^a^	9.999 ± 0.487^a^	7.756 ± 0.177^b^
	Solubility (%)			
7 days	26.53 ± 1.331^a^	20.07 ± 2.385^b^	20.92 ± 1.163^b^	-------
14 days	29.77 ± 2.646^a^	20.42 ± 1.757^b^	20.78 ± 0.943^b^	-------

CH-EXP, experimental calcium hydroxide paste; CH-F18BG, calcium hydroxide + F18 bioactive glass; CH-Cu/F18BG, calcium hydroxide + copper-doped F18 bioactive glass; Control, distilled water.

Different superscript letters in the same line indicate statistically significant differences among the groups (p<0.05).

The results of antimicrobial activity are shown in Tables 3 and 4. In the single-species *E. faecalis* biofilm model, the calcium hydroxide formulation combined with copper-doped F18 bioactive glass promoted the greatest reduction in bacterial counts (p<0.05). The formulations containing calcium hydroxide with F18 bioactive glass and the experimental calcium hydroxide paste showed similar results (p>0.05), but all experimental groups performed better than the control (p<0.05). In the dual-species biofilm model (*E. faecalis* and *C. albicans*), the formulation with copper-doped F18 bioactive glass promoted the greatest reduction in *E. faecalis* (p<0.05). The experimental calcium hydroxide paste completely eliminated C. albicans (p<0.05). In addition, the formulation with copper-doped F18 bioactive glass was significantly more effective against *C. albicans* than the formulation with F18 bioactive glass (p<0.05), and both outperformed the control (p<0.05).

**Table 3:** Mean and standard deviation (±SD) of the modified direct contact test (log10 CFU/mL) in the single-species *E. faecalis* biofilm after 15 h of contact for the experimental groups and the control

CH-EXP	CH-F18BG	CH-Cu/F18BG	Control
5.596 ± 0.214^a^	5.141 ± 0.775^a^	3.952 ± 0.764^b^	7.985 ± 0.248^c^

CH-EXP, experimental calcium hydroxide paste; CH-F18BG, calcium hydroxide + F18 bioactive glass; CH-Cu/F18BG, calcium hydroxide + copper-doped F18 bioactive glass; Control, distilled water.

Different superscript letters in the same line indicate statistically significant differences among the groups (p<0.05).

**Table 4:** Mean and standard deviation (±SD) of the modified direct contact test (log10 CFU/mL) in the dual-species *E. faecalis* and *C. albicans* biofilm after 6 h of contact for the experimental groups and the control

Microorganism	CH-EXP	CH-F18BG	CH-Cu/F18BG	Control
*E. faecalis*	3.545 ± 0.174^a^	3.68 ± 0.407^a^	2.977 ± 0.416^b^	6.617 ± 0.222^c^
*C. albicans*	0.00 ± 0.00^a^	4.387 ± 0.496^b^	2.764 ± 0.422^c^	6.541 ± 0.319^d^

CH-EXP, experimental calcium hydroxide paste; CH-F18BG, calcium hydroxide + F18 bioactive glass; CH-Cu/F18BG, calcium hydroxide + copper-doped F18 bioactive glass; Control, distilled water.

Different superscript letters in the same line indicate statistically significant differences among the groups (p<0.05).

Table 5 summarizes the bond strength (push-out) values. For AH Plus, no significant differences were observed among the intracanal medications evaluated (p>0.05). For Bio-C Sealer, the group previously exposed to calcium hydroxide combined with F18 bioactive glass showed significantly higher bond strength values than those observed under the other conditions (p<0.05). When comparing the sealers, AH Plus exhibited greater bond strength than Bio-C Sealer in all groups, regardless of the intracanal medication used (p<0.05).

**Table 5:** Mean bond strength (MPa) for AH Plus and Bio-C Sealer after prior contact with different intracanal medications and the control

Sealer	CH-EXP	CH-F18BG	CH-Cu/F18BG	Control
AH Plus	11.5305^Aa^	12.7548^Aa^	12.143^Aa^	11.8105^Aa^
Bio-C Sealer	6.0632^Ab^	8.9569^Bb^	6.0537^Ab^	3.0798^Cb^

CH-EXP, experimental calcium hydroxide paste; CH-F18BG, calcium hydroxide + F18 bioactive glass; CH-Cu/F18BG, calcium hydroxide + copper-doped F18 bioactive glass; Control, no intracanal medication.

Lowercase superscript letters in the same column indicate statistically significant differences between the sealers (p<0.05). Uppercase superscript letters in the same line indicate statistically significant differences among the intracanal medications (p<0.05).

Table 6 shows the distribution of failure modes observed after the bond strength test. For AH Plus, the cohesive pattern was the most frequent in all groups. For Bio-C Sealer, mixed failure predominated in the experimental calcium hydroxide group, the calcium hydroxide combined with copper-doped F18 bioactive glass group, and the control group. In the calcium hydroxide combined with F18 bioactive glass group, the predominant pattern was cohesive.

**Table 6:** Percentage distribution (%) of failure modes in the experimental groups and the control in the push-out bond strength test of AH Plus and Bio-C Sealer

Sealer	Failure mode	CH-EXP	CH-F18BG	CH-Cu/F18BG	Control
AH Plus	Mixed	33%	-	-	33%
	Adhesive	-	-	14%	22%
	Cohesive	67%	100%	86%	45%
Bio-C Sealer	Mixed	67%	33%	50%	42%
	Adhesive	33%	-	30%	25%
	Cohesive	-	67%	20%	33%

CH-EXP, experimental calcium hydroxide paste; CH-F18BG, calcium hydroxide + F18 bioactive glass; CH-Cu/F18BG, calcium hydroxide + copper-doped F18 bioactive glass; Control, no intracanal medication.

A dash (-) indicates absence of that failure mode.

## Discussion

Calcium hydroxide-based intracanal medications are widely used due to their recognized antimicrobial potential and biocompatibility. Modification of calcium hydroxide-based intracanal medication by incorporating vehicles and additives may increase antimicrobial activity, bioactive potential, and optimize the physicochemical properties of the material ^9^ ^17^ ^23^. In this context, the combination with bioactive glasses, such as F18 bioactive glass and its copper-doped form, emerges as a promising strategy. In the present investigation, the incorporation of bioactive glasses into calcium hydroxide reduced solubility, increased bond strength, and enhanced antimicrobial activity. Consequently, the null hypothesis was rejected.

In the present study, all experimental pastes promoted alkalinization of the medium at all evaluated periods. This finding confirms the ion-releasing capacity of the formulations. Similar results were reported for an experimental cement based on 45S5 bioactive glass, which maintained an alkaline pH for up to 15 days ^22^. Similarly, calcium hydroxide pastes maintained high pH values for up to 21 days ^17^. The increase in pH is associated with the release of hydroxyl ions, the mechanism responsible for the antimicrobial action and biological effects of calcium hydroxide ^2^. In bioactive glasses, the pH increase results from ionic exchange, with calcium and sodium released in exchange for hydrogen ions ^23^ ^24^.

The replacement of distilled water after each experimental period during pH analysis is carried out to allow the evaluation of the ion release capacity in each experimental period ^9^ ^17^. The use of distilled water allows the evaluation of ion release by the materials, although it does not reproduce physiological conditions, such as the use of simulated body fluid (SBF) ^(25)^. The solubility of endodontic materials is traditionally evaluated by mass loss after immersion in an aqueous medium ^9^ ^17^. In the present study, the experimental calcium hydroxide-based paste showed greater solubility compared with the formulations combined with F18 bioactive glass and copper-doped F18 bioactive glass. This result may be associated with the bioactive behavior of bioactive glass; upon contact with aqueous fluids, it promotes ionic exchange reactions with the release of calcium and phosphate ions. These ions favor the nucleation and growth of hydroxyapatite crystals on the material surface ^26^. The deposition of this mineral deposit may have increased the surface mass of the formulations, thereby explaining the lower solubility values observed for the pastes containing bioactive glass compared with the experimental paste.

In this study, the antimicrobial activity of the formulations was evaluated using the modified direct contact test ^20^. The single-species biofilm consisted of *E. faecalis*, whereas the dual-species biofilm included *E. faecalis* associated with *C. albicans*, microorganisms frequently linked to the persistence of endodontic infections ^27^. Bovine dentin has been used as a substrate for biofilm development due to its availability and structural similarity to human dentin ^28^. However, differences in dentinal tubules between bovine and human dentin may influence the interaction between materials and the dentin substrate ^29^.

The use of eluates was chosen to standardize the release of soluble components from the formulations, enabling reproducibility, as previously described ^30^ ^31^. Furthermore, direct contact between paste and bacterial inoculum hinders the separation between intracanal medication residues and microorganisms during microbiological plating, potentially interfering with microbiological results ^31^. However, this model does not completely reproduce the clinical conditions of direct contact between intracanal medications and biofilms within the root canal system.

The results showed that the calcium hydroxide formulation combined with copper-doped F18 bioactive glass exhibited greater efficacy against *E. faecalis* in both models. Similar findings were reported in a study that demonstrated larger inhibition zones on agar for the combination of calcium hydroxide with copper nanoparticles compared with calcium hydroxide alone ^32^. The antimicrobial effect of calcium hydroxide is attributed to the increase in pH resulting from the release of hydroxyl ions, which creates an unfavorable environment for microbial growth ^2^. Copper further enhances this effect through different mechanisms, such as the generation of reactive oxygen species, oxidative stress, and lipid peroxidation, which compromise cell membrane integrity ^33^. These effects also include protein oxidation and bacterial DNA damage ^34^ ^35^. Therefore, the mechanisms attributed to copper appear to act in a complementary manner to calcium hydroxide, which may explain the enhanced antibacterial effect observed in the present investigation.

The current findings demonstrated that the experimental calcium hydroxide-based paste completely eliminated *C. albicans* in the dual-species biofilm model, showing a significant difference from the other formulations. Although all experimental groups showed similar alkaline pH values, calcium hydroxide may have exerted a direct antifungal effect in the absence of bioactive glass particles. The incorporation of bioactive glass may have altered the ion release kinetics, diffusion dynamics, or the interaction between the drug and the biofilm matrix, potentially modulating antifungal activity ^36^. A previous study demonstrated that metallic nanoparticles do not necessarily increase antifungal activity against C. albicans when compared to calcium hydroxide alone ^37^. Furthermore, C. albicans is highly sensitive to alkaline environments, since calcium hydroxide raises the pH of the medium to levels incompatible with fungal survival and inhibits its morphological transition to the hyphal form, which is essential for virulence ^2^ ^38^. In mixed biofilms, *C. albicans* also undergoes greater metabolic pressure due to competition with *E. faecalis*, which may compromise its adaptation and resistance ^38^ ^39^. Taken together, these factors may explain the complete elimination of *C. albicans* observed only in the group treated with the experimental paste. Therefore, the antifungal effect observed for the experimental calcium hydroxide paste was likely multifactorial and cannot be attributed exclusively to alkalinity.

The bond strength between endodontic sealers and root dentin plays a key role in ensuring the integrity of the material-dentin interface. This property is essential to prevent microbial leakage ^21^. Intracanal medications may interfere with this process, either enhancing or compromising the adhesion of filling materials ^21^. In the present investigation, AH Plus exhibited significantly higher bond strength values than Bio-C Sealer, regardless of the intracanal medication. These results corroborate previous studies that highlighted the superior performance of AH Plus compared with calcium silicate-based sealers ^40^ ^41^. The difference may be related to the chemical composition of the sealers and the type of interaction established at the material-dentin interface ^40^. AH Plus, an epoxy resin-based sealer, is capable of forming chemical interactions with dentin collagen. This process occurs through the reaction of epoxy rings with amino groups of collagen fibers in the dentin matrix, resulting in covalent bonds ^42^. This mechanism explains the stronger adhesion of AH Plus compared with the bioceramic sealer Bio-C Sealer.

Regarding Bio-C Sealer, the current findings showed that prior exposure to intracanal medications enhanced bond strength to dentin. The effect was more evident when calcium hydroxide was combined with F18 bioactive glass, which yielded the highest values. This outcome may be related to the presence of ZrO₂ in both the experimental formulations and Bio-C Sealer. Although often described as inert, the literature indicates that ZrO₂ can increase the bioactivity of calcium silicate-based cements ^7^ ^43^. This component can contribute to the deposition of hydroxyapatite crystals, reinforcing the interaction with dentin ^43^ and potentially improving the sealing capacity of the material-dentin interface. In addition, partial replacement of Portland cement with 30% ZrO₂ has been associated with greater calcium ion release. These ions react with phosphates in simulated fluids to form calcium phosphate, thereby enhancing the bioactivity of the material ^7^.

In this context, the presence of F18 bioactive glass may have acted synergistically. When in contact with aqueous fluids, it releases sodium and calcium ions, promotes the formation of a silica gel layer, and induces the precipitation of amorphous calcium phosphate, which subsequently transforms into hydroxyapatite ^8^ ^26^ ^44^. This bioactive process may favor the integration between Bio-C Sealer and root dentin. This could explain the higher bond strength values observed for this combination. However, the clinical relevance of this finding should be interpreted with caution, since higher bond strength values ​​do not necessarily guarantee better sealing capacity or less microleakage under clinical conditions ^45^. The push-out test represents an indirect measure of sealing capacity and a laboratory model that does not fully reproduce the mechanical, biological and chemical challenges present in the root canal system over time ^46^. Therefore, future studies evaluating interfacial adaptation, dentinal tubule penetration and microleakage under clinical conditions are needed to confirm the clinical relevance of these findings.

The analysis of failure modes in this investigation reinforced the different behavior between the tested sealers. For AH Plus, the cohesive pattern predominated in all groups. This finding reflects stable adhesion to dentin, as previously reported ^47^. In contrast, Bio-C Sealer showed greater variability, with mixed failures being the most frequent. The exception occurred when Bio-C Sealer was combined with F18 bioactive glass, in which the cohesive pattern was more prevalent. This finding suggests that the incorporation of F18 bioactive glass may favor the interaction between the bioceramic sealer and dentin, corroborating the superior bond strength observed in this study.

The findings of this research suggest that the incorporation of bioactive glass into calcium hydroxide may optimize intracanal formulations. Copper-doped F18 bioactive glass demonstrated superior antimicrobial effectiveness against *E. faecalis*, while both bioactive glass formulations reduced solubility and maintained an alkaline pH. Regarding bond strength, the most pronounced effect was observed for Bio-C Sealer after prior contact with calcium hydroxide combined with F18 bioactive glass. These results highlight the potential of bioactive glass, in its different forms, as an adjuvant in intracanal medications, extending the benefits beyond antimicrobial action and favoring the material-dentin interaction.

The limitations of the present study should be considered. The in vitro design does not fully reproduce the complexity of clinical conditions. In addition, bovine dentin was used as substrate, and its differences from human dentin in tubule density and permeability may limit clinical extrapolation. The antimicrobial assay was based on eluates, which improves standardization but does not simulate the direct contact of a dense paste with biofilm inside the root canal. Moreover, biofilm structural analysis, ion release quantification, cytotoxicity assessment, and evaluation of paste removability before obturation were not performed. Therefore, future studies using more clinically relevant models and complementary analyses are necessary to better clarify the translational potential of these formulations.

In conclusion, the calcium hydroxide formulation combined with F18 bioactive glass reduced solubility and enhanced the bond strength of the bioceramic sealer Bio-C Sealer. The formulation with copper-doped F18 bioactive glass improved antimicrobial activity, particularly against *E. faecalis*. These findings reinforce the potential of these formulations as promising intracanal medications, providing advantages beyond antimicrobial effects and opening perspectives for future applications in endodontics.

## Data Availability

The data that support the findings of this study are available from the corresponding author upon reasonable request.
